# Supplementary figures and images for: Impact of Covid -19 incidence rate and government-initiated risk communication measures on individual’s NPI practices
Source: PLoS One. 2024 Mar 14;19(3):e0283294. doi: 10.1371/journal.pone.0283294 (PMC10939235; doi:10.1371/journal.pone.0283294)

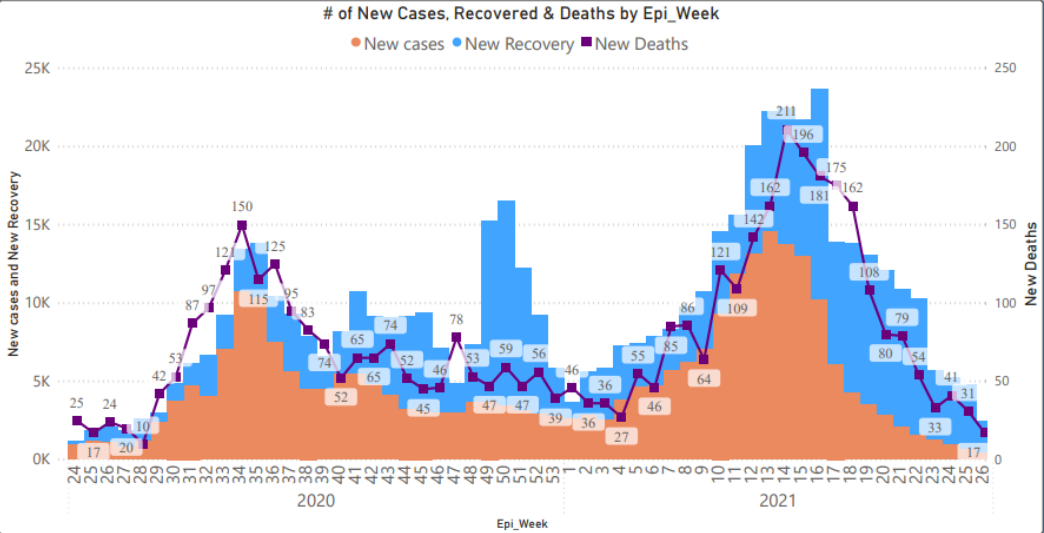
 **S1_raw_image. COVID-19 confirmed cases, recovery and death by Epi-Week as of July 04, 2021, Ethiopia**

Supplement: S1 Raw image — (DOCX) [file pone.0283294.s001.docx]
