# Supplementary material for: Impact of Covid -19 incidence rate and government-initiated risk communication measures on individual’s NPI practices
Source: PLoS One. 2024 Mar 14;19(3):e0283294. doi: 10.1371/journal.pone.0283294 (PMC10939235; doi:10.1371/journal.pone.0283294)
Supplement: Attachment — Submitted filename: Response to Reviewers.docx [file pone.0283294.s007.docx]

| **Editor and Reviewers comments** | **Authors response** |
| --- | --- |
| **By Editor comment** |  |
| 1. Please ensure that your manuscript meets PLOS ONE's style requirements, including those for file naming. The PLOS ONE style templates can be found at  https://journals.plos.org/plosone/s/file?id=wjVg/PLOSOne_formatting_sample_main_body.pdf and | We accepted the comment and revised the manuscript following the PLOS ONE guideline |
| 2. Please provide additional details regarding participant consent. In the ethics statement in the Methods and online submission information, please ensure that you have specified (1) whether consent was informed and (2) what type you obtained (for instance, written or verbal, and if verbal, how it was documented and witnessed). If your study included minors, state whether you obtained consent from parents or guardians. If the need for consent was waived by the ethics committee, please include this information.  If you are reporting a retrospective study of medical records or archived samples, please ensure that you have discussed whether all data were fully anonymized before you accessed them and/or whether the IRB or ethics committee waived the requirement for informed consent. If patients provided informed written consent to have data from their medical records used in research, please include this information | We provided detailed information about ethical processes followed and attached the IRB letter. |
| 3. We suggest you thoroughly copyedit your manuscript for language usage, spelling, and grammar.  Upon resubmission, please provide the following:  The name of the colleague or the details of the professional service that edited your manuscript  A copy of your manuscript showing your changes by either highlighting them or using track changes (uploaded as a *supporting information* file)  A clean copy of the edited manuscript (uploaded as the new *manuscript* file) | Comment accepted. The manuscript was language edited. Both a track change and a clean copy of the manuscript are uploaded. |
| 4. Thank you for stating the following in the Acknowledgments Section of your manuscript:  “We would like to thank the Federal Democratic Republic of Ethiopia Ministry of Health and the College of Health Sciences and the School of Public Health of Addis Ababa University for supporting this study.”  We note that you have provided additional information within the Acknowledgements Section that is not currently declared in your Funding Statement. Please note that funding information should not appear in the Acknowledgments section or other areas of your manuscript. We will only publish funding information present in the Funding Statement section of the online submission form. Please remove any funding-related text from the manuscript and let us know how you would like to update your Funding Statement. Currently, your Funding Statement reads as follows:  “The author(s) received no specific funding for this work.” | We corrected accordingly |
| 5. We note that Figure 1 in your submission contain map images which may be copyrighted. All PLOS content is published under the Creative Commons Attribution License (CC BY 4.0), which means that the manuscript, images, and Supporting Information files will be freely available online, and any third party is permitted to access, download, copy, distribute, and use these materials in any way, even commercially, with proper attribution. For these reasons, we cannot publish previously copyrighted maps or satellite images created using proprietary data, such as Google software (Google Maps, Street View, and Earth). For more information, see our copyright guidelines: <http://journals.plos.org/plosone/s/licenses-and-copyright>. | Comment accepted. The map was taken from the Ethiopian Statistical Agency website and is available in the public domain. |
| **Reviewer 1 comments** |  |
| INTRODUCTION 1) The article introduction states that the “Federal Ministry of Health's (FMoH) Ethiopian Public Health Institute (EPHI) takes the lead in coordination with regional partners and governments." Provide more context on the level of heterogeneity of policies and interventions across Ethiopia. Please expand the introduction to explain if regions and cities have authority to implement their own local NPI policies. Did religious locations, businesses also implement or recommend their own NPI policies that could influence the compliance at observation locations associated with their facility? | Comment accepted. Detail information about regional and federal level EPHI roles and responsibilities is included in Lines, #100-12 |
| 2) The introduction should provide an overview of policies, interventions, and awareness campaigns. For example, The overview of the 76 WHO interventions in the Discussion section could be moved to the introduction. Please include a brief description of the key public health policy intervention changes where the Discussion infers they have influence on NPI compliance. Examples of inference from the Discussion include the Ethiopian State of Emergency, and increased community compliance level starting in week 26. Also please provide with relevant beginning and ending dates. (Please see comment on Method on trend and temporal claims) | Comment accepted. WHO documented national Covid-19 prevention interventions were summarized. The total number of interventions during the monitoring period was corrected to 59. The details presented in Lines, #74-9 and #100-6 |
| METHODS/CONCLUSION 3) The Methods section should be updated to include a statistical analysis of trends over time. The trend lines in Figures 1 and 2 are not sufficient to support the conclusion. For example, "Proper hand hygiene" and "Proper physical distancing" visually appear to be declining until the break in data collection, but not after the break. Also the respiratory hygiene lines in Figures 1 and 2 do not appear to decline. | Comment accepted. The method section is updated in Lines #188-95 |
| 4) The conclusions claim that the implementation of government-initiated public measures followed by an increased observation of NPI compliance is not supported by methods. To support this claim the Methods section should be updated to include an approach to statistically test for pre-post policy and intervention changes. As noted above, the introduction should clearly explain the timing and scope of any intervention that the authors claim influenced NPI compliance. | Comment accepted. The conclusion is revised and supportive information is added in the introduction and the methods sections in lines #78-9, #85-95, #100-13, #130-32, #311-20, #322-7, #331-41 |
| 5) Please explain the subject selection process in more detaiil. This is important because the proportion of age and gender in the population from which subjects were selected may have changed over the course of the data collection. More detail on the process to randomly select individuals can help the readers understand measurement variations over the duration of the study. For example, the proportion of observations of people less than age 18 increases over the first 17 weeks. A detailed explanation of subject selection will help the reader understand if the variation reflects a change in population from which subjects are selected or is due to the selection process itself. | Comment accepted. Participant selection process added in lines #166-72. |
| 6) Provide an explanation for the interrupted data collection in the 27th and 28th weeks. This interruption coincided with increased compliance observed in both Addis Ababa and regional cities around the 26-30th weeks. | Comment accepted. The reason for the data collection interruption was added in lines #173-6 |
| RESULTS 7) Table 1, please update the manuscript to comment on the drop in observations count beginning during 17th week (Jan 25-31). For example, was change due to a change in the study or does the reduced number of observations coincide with a reduced population at the observation locations. | Comment accepted. The reason for the reduced number of observations is described in the lines #173-6 |
| DISCUSSION, CONCLUSION 8) Update this section after Introduction and Methods edits to reflect the changes. | Comment accepted. The discussion part is entirely revised. |
| LIMITATIONS 9) Please expand on limitations. The manuscript references limitations from a prior study of much smaller scope and different time period. Examples of new limitations could include changes of population mix in the observed locations over the life of the study, unknowns of the impact of the 76 WHO interventions, and recommendations for interpreting results outside of Ethiopia. | Comment accepted. Additional information included in lines #384-7 |
| STROBE attachment: 10) The STROBE checklist should reflect revisions to the main manuscript.  General: Please review for grammar. | The STROBE checklist was revised accordingly and the manuscript was language edited. |
| **Reviewer 2** |  |
| Reviewer #2: 1. The title of the study and the conclusion, which focused on public initiated NPIs and their impact are not the major study findings and requires revision. The study findings were neither linked to government initiated nor public initiated NPI measures. It only presented the trends. Hence the conclusion does not reflect the findings of the study. | Comment accepted. The title is revised in this version as “Impact of Covid -19 incidence rate and government-initiated risk communication measures on Individual NPI Practices”. |
| 2. The study lacks clear definition/distinction between government initiated and public initiated NPIs use. The government started promoting NPI use for COVID 19 prevention through media and other platforms early on. Eg School closure was enforced following the first case detection in March 2020. By the time you started tracking NPIs compliance, the MOH and EPHI had been promoting NPI use through different platforms, whereby RCC was one of the strategies. Government-initiated NPI interventions are expected to be practiced and maintained by the public with good compliance to get good effect. But this does not make it public initiated. | Comment accepted. The government-initiated measures were corrected including in the title. |
| 3. It is very natural that any behavioral interventions showed high or low adherence overtime. Describing only the ups and downs using timeline have little value. Please relate the changing trend with enforced government prevention strategies, case and death detection and reporting, disease progression and burden, public panic… | Comment accepted. Additional review information is included in the introduction, method, and discussion sections at different lines #78-9, #85-95, #100-13, #130-32, #311-20, #322-7, #331-41 |
| 4. More contextual information is required in the methods section about study sites. Eg Regional capital cities are confusing. | Comment accepted. Brief information is added about the context of regional cities in lines #100-12, #138-47 |
| 5. The manuscript benefit from language editing. Some comments are given on manuscript here attached. | Comment accepted and the manuscript was language edited |
