# Supplementary material for: Impact of Covid -19 incidence rate and government-initiated risk communication measures on individual’s NPI practices
Source: PLoS One. 2024 Mar 14;19(3):e0283294. doi: 10.1371/journal.pone.0283294 (PMC10939235; doi:10.1371/journal.pone.0283294)
Supplement: Attachment — Submitted filename: Response to Reviewers.docx [file pone.0283294.s008.docx]

| **Reviewer’s comments** | **Authors response** |
| --- | --- |
| 1. Study aims and methods do not support showing how population NPI compliance is related to government-initiated risk communication measures. Although Introduction and Discussion do mention important government NPI actions, the Results section does not mention risk communications occurrences along the observational timeline. Also, Figures do not contain risk communication initiatives time points with the NPI compliance observations. The Figure S1_raw_image does show case and death rates, but not in relation to NPI observations. Based on results and figures provided, readers cannot link government intervention with NPI observations. Therefore, the following content is not supported by the current methods and results: • Title: “government-initiated risk communication measures”. • Lines 26-27: “relation to Covid-19 incidence and government-initiated interventions” • Line 131: “determining the trend of the weekly changes in the three individual-level NPI behaviors and its relationship with the incidence rate…”. Results and Figures do not show incident rate in separate figure, not in relation to NPI behaviors. • Line 131: “and government-initiated measures.” Introduction and Discussion do include government initiated measures, but Results and Figures do not show government-initiated measures in relation to NPI behaviors. • Line 390: Linkage in Figures and Results between timing of government-initiated measures should be made before justifying the following comment in the conclusion: “Community adherence to NPI practices was not sustainable after the decline in incidence and lack of new interventions.” | Comments accepted.  A new analysis graph, Figure 2 added in the result section which contains the NPI trends, Covid-19 incidence and public health measures timeline. Accordingly, changes made in the Abstract, method, result and conclusion section to include the new result presentations |
| 2. A new Results table showing beginning and end dates of the government interventions mentioned in the Introduction in relation to the “WHO Epi week *” would be helpful to future researchers that use the study data set. The study indicates there are national and many regional NPI policies. A table with policies that authors believe are most important would be sufficient. | The information is included in the new Figure 2 |
| Minor 3. Lines 46, 336, Awkward grammar, please rephrase: “compliance did not sustain longer”. | Commented accepted and corrected |
| 4. Line 137: In Methods, study seems to be an unobtrusive observation with repeated measures and descriptive results, not a “cross-sectional study design”. Please update study design description that more accurately describes the methods. | Thank you actually, the study is "Repeated cross-sectional Observations" which implies just repeating the cross-sectional observation over time. Of course, the findings are descriptive. |
| 5. Line 170: Please clarify that the data collection tool controls the random subject selection and not the observer. It is important with many geographically scattered observers that randomization is not subject to individual observer bias. | Commented accepted and described |
| 6. Line 209, 319: Please state the reason “observation was halted for two weeks”. It is understandable that this interruption happened, but authors need to explain the reason. For example, government curfews or lock downs prevented observers from performing their tasks. | Commented accepted and described |
| 7. Line 195: Methods section mentions “appropriate hand hygiene”. File S2, Annex 1: “COVID-19 Prevention Practices Data Collection Tool in a community” lists both hand sanitization and hand washing. Please clarify if “hand hygiene” in the manuscript requires either or both to be considered “appropriate hand hygiene”. | Commented accepted, definition included |
