# Supplementary material for: Impact of Covid -19 incidence rate and government-initiated risk communication measures on individual’s NPI practices
Source: PLoS One. 2024 Mar 14;19(3):e0283294. doi: 10.1371/journal.pone.0283294 (PMC10939235; doi:10.1371/journal.pone.0283294)
Supplement: Attachment — Submitted filename: Response to Reviewers.docx [file pone.0283294.s009.docx]

| **Editor comment** | **Authors response** |
| --- | --- |
| What do the “^¶”^ and “^&”^ you put with the author’s name stands for? Please describe them if they show a contribution or other information, or remove them if not necessary. | Comment accepted, deleted |
| Please review your reference list to ensure that it is complete and correct. If you have cited papers that have been retracted, please include the rationale for doing so in the manuscript text, or remove these references and replace them with relevant current references. Any changes to the reference list should be mentioned in the rebuttal letter that accompanies your revised manuscript. If you need to cite a retracted article, indicate the article’s retracted status in the References list and also include a citation and full reference for the retraction notice. | 1. The title for all list of reference were italic now changed 2. Minor edition on the reference lists of the World Health Organization (#1,7,9,9,32,&35) corrected. |
| **Reviewer comment** |  |
| No reviewer comment and accepted | Thank you |
